# Electromagnetic Fenton Combined with Electro-Biological Coupling Technology for Treating High-Nitrogen Organic Chemical Wastewater

**DOI:** 10.3390/toxics13121059

**Published:** 2025-12-06

**Authors:** Dengyan Mu, Xiaojie Chen, Peiyu Zhao, Houhui Zhang, Zhujun Bai, Baoshan Wang

**Affiliations:** 1 Gansu Youyuan Environmental Protection Engineering Technology Co., Ltd., Lanzhou 730030, China; 18693174816@163.com; 2 School of Environmental and Municipal Engineering, Lanzhou Jiaotong University, Lanzhou 730070, China; 12221154@stu.lzjtu.edu.cn (P.Z.); 11240152@stu.lzjtu.edu.cn (Z.B.); 3 Shaanxi Key Laboratory of Environmental Engineering, Xi’an University of Architecture and Technology, Xi’an 710055, China; xauatchenxj@163.com; 4 Gansu Appraisal Center for Ecological Environment & Engineering, Lanzhou 730030, China; zhanghouhui04@163.com

**Keywords:** high-nitrogen wastewater, electro-magnetic Fenton, 3D biofilm electrode reactor, autotrophic denitrification, advanced oxidation

## Abstract

High-nitrogen organic chemical wastewater is characterized by high chemical oxygen demand (COD_Cr_), poor biodegradability, and toxic nitrogenous organics, posing significant challenges for conventional biological treatment. In this study, a dual-electrical treatment strategy integrating an electromagnetic Fenton (EM-Fenton) pretreatment unit with a three-dimensional biofilm electrode reactor (3D-BER) is proposed. The EM-Fenton system used iron–carbon fillers under electric and magnetic fields to generate hydroxyl radicals (·OH), enabling efficient oxidation of nitro-aromatic compounds and the conversion of organic nitrogen into NO_3_^−^-N, while reducing Fe^2+^ input and iron sludge generation. Subsequently, the 3D-BER, filled with Fe_3_O_4_/Mn_3_O_4_-modified polyurethane spheres, facilitated autotrophic denitrification and phosphorus removal through enhanced extracellular electron transfer and trace hydrogen (H_2_) release. Experimental results demonstrated that the EM-Fenton system achieved COD_Cr_ and NH_4_^+^ removal rates of over 40% and 14%, respectively, under optimal HRT. The 3D-BER further improved removal efficiencies, with TN and TP reductions exceeding 80% and 81%, respectively, significantly outperforming the control groups. Microbial analysis revealed the enrichment of functional genera, such as *Pararhodobacter* and *Thauera*, and the upregulation of key denitrification pathways. This coupled system demonstrated high treatment efficiency, process synergy, and microbial selectivity, offering a promising approach for the advanced treatment of high-nitrogen industrial wastewater.

## 1. Introduction

The continuous discharge of high-concentration-nitrogen-containing organic wastewater is leading to increasingly severe global water pollution across various industries, such as petrochemicals, coal chemicals, pharmaceuticals, dyes, daily chemicals, non-ferrous metal hydrometallurgy, and leachate treatment [1]. Statistics indicate that seven major industries, particularly chemical companies [2], are responsible for approximately 70% of global freshwater utilization and pollution. Additionally, regional differences in economic development pose further water resource management challenges. In economically underdeveloped areas, insufficient industrial wastewater treatment capacity leads to the direct discharge of toxic substances into water bodies, posing a serious threat to aquatic ecosystems and human health [3]. This type of wastewater is typically characterized by large fluctuations in water quality and volume, high COD_Cr_ and total nitrogen (TN) concentrations, enrichment of recalcitrant organic compounds (such as heterocyclic compounds and long-chain alkanes), and strong biological toxicity. The ratio of biochemical oxygen demand to chemical oxygen demand (B/C) is generally below 0.2, indicating extremely poor biodegradability [3,4]. Generally, when treating wastewater in water treatment plants, anaerobic pre-treatment is performed first. Although this process can remove most of the organic matter, the effluent C/N often drops below 0.5, and the concentration of ammonia nitrogen (NH_4_^+^-N) can suddenly rise to 240–400 mg/L. This leads to low denitrification efficiency in traditional nitrification and denitrification processes owing to carbon source scarcity, ultimately resulting in excessive emissions of pollutants such as TN [5]. It is worth noting that excessive nitrogen pollutants entering water bodies not only cause eutrophication, but their oxidation products, nitrate (NO_3_^−^-N) and nitrite (NO_2_^−^-N), also pose threats to human health through the food chain, while disrupting the balance of aquatic ecosystems and leading to a sharp decline in biodiversity [6,7].

Current nitrogen-containing wastewater treatment technologies are mainly categorized as physical, chemical, and biological categories. Although physical and chemical methods, such as blow-off, magnesium ammonium phosphate (MAP) precipitation, and chemical oxidation, are suitable for the pretreatment of wastewater containing high concentrations of toxic nitrogen, they have significant drawbacks, such as a the high risk of secondary pollution and incomplete total nitrogen removal [7]. In contrast, biological denitrification technologies, including anaerobic ammonification processes such as UASB, IC, and EGSB, as well as nitrification denitrification processes such as A/O, SBR, and SMBR, are widely used because of their low operating costs and environmental friendliness [8]. However, when treating low-C/N (<0.5) wastewater, these technologies encounter bottlenecks in denitrification efficiency due to insufficient carbon sources. Heterotrophic denitrifying bacteria require a substantial amount of organic carbon to convert NO_3_^−^-N, and the residual recalcitrant organic matter in the wastewater cannot be effectively utilized, resulting in a technical challenge of carbon–nitrogen supply demand imbalance. In response to the limitations of traditional processes for treating high-nitrogen organic wastewater, a new technology based on the coupling of advanced oxidation and biological treatment has emerged as a research hotspot. Electro-Fenton technology enhances the Fe^2+^/Fe^3+^ cycle through high-frequency electromagnetic fields, enabling the generation of reactive oxygen species (such as hydroxyl radical) and achieving the chain-breaking of recalcitrant organic compounds [9,10]. Electro-biological coupling technology utilizes the electrochemical driving effect of an three-dimensional biofilm electrode reactor (3D-BER) to promote the synergistic metabolism of autotrophic and heterotrophic microorganisms and enhance nitrogen conversion without an external carbon source [11].

The electromagnetic Fenton (EM-Fenton) combined with electro-biological coupling technology proposed in this study aims to overcome the carbon source limitation in low C/N wastewater treatment through a synergistic mechanism of “chemical oxidation pretreatment electric-driven biological denitrification,” providing theoretical and technical support for the efficient treatment of high-nitrogen organic chemical wastewater.

## 2. Methods

### 2.1. Overview of High-Nitrogen Wastewater Quality

High-nitrogen wastewater, sourced from actual wastewater in a chemical industrial park, exhibits complex water quality, high organic matter concentration, and a certain degree of toxicity. Therefore, pretreatment is necessary to remove COD_Cr_ and increase BOD_5_ to enhance the advantages of the subsequent 3D-BER. As shown in Table 1, the second row represents the raw water quality, and the third row represents the inlet water quality of the 3D-BER, which was configured in a 3:1 ratio between of EM-Fenton-water to domestic sewage.

### 2.2. Material Design and Device Design

**Figure 1:**
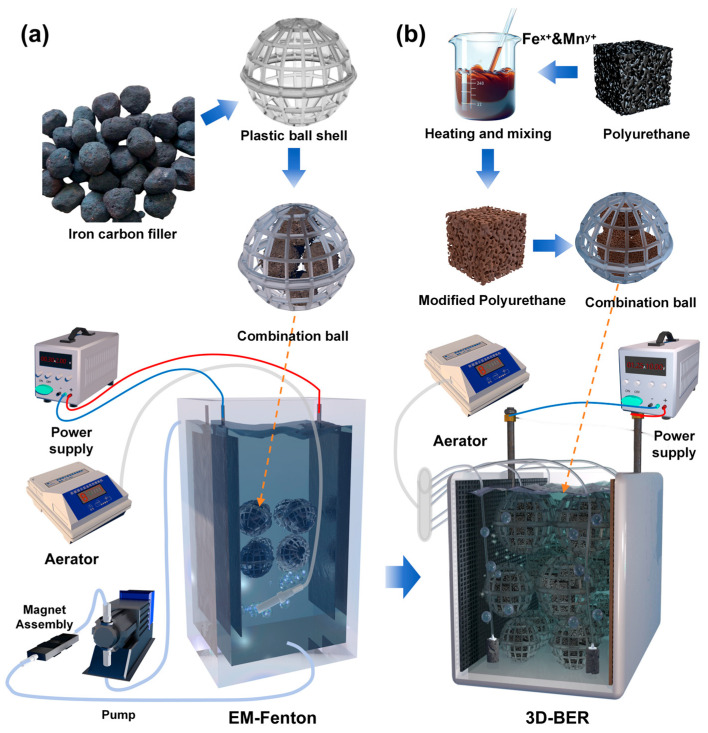
(**a**) Preparation and device design diagram of the electromagnetic Fenton filler; (**b**) Device diagram of the modified polyurethane filler and the three-dimensional biofilm electrode reactor. Reproduced or adapted from [12], with permission from Elsevier 2025.

Due to the complex water quality, high COD_Cr_, high N content, and a high proportion of organic nitrogen in the initial high-nitrogen organic chemical wastewater, two types of fillers were designed and modified in this study: an iron-carbon particle filler ball Figure 1a and a modified polyurethane filler ball in Figure 1b. The iron-carbon particle electrode was a purchased commodity filler and was wrapped in a plastic shell with a diameter of 30 mm. The polyurethane-modified filler was made from water-based polyurethane with iron oxide and manganese oxide as the core components. The original polyurethane (PU) carrier was a commercial product purchased from Yixing Shengquan Water Treatment Equipment Co., Ltd. (Yixing, Jiangsu, China), and used as received without further purification. The Fe_3_O_4_/Mn_3_O_4_-modified PU spheres employed in the 3D-BER were prepared in our laboratory by loading iron and manganese oxides onto this commercial PU carrier according to the procedure described below. It was prepared in a 20:1:5 ratio under heating in an 80 °C water bath, and dried in an oven at 60 °C for subsequent use. During the subsequent 3D-BER start-up, the middle was filled as a particle electrode, acting as the third electrode.

The main body of the EM Fenton device was constructed using an organic glass plate (L × B × H = 20 cm × 15 cm × 20 cm). Four rubidium permanent magnets (4 × 5 cm) were symmetrically mounted on the upper and lower outer walls of the EM-Fenton reactor (two on the top and two directly opposite on the bottom), forming a stable transverse static magnetic field of approximately 50 mT (±10 mT) at the inner reactor wall. The detailed magnet arrangement is shown in Figure S1 in the Supporting Information. An electric field was applied using a DC regulated power supply connected to a ruthenium–iridium anode and a titanium cathode (plate spacing 15 cm), operated at 8.0 V and 0.6 A. A peristaltic pump was used for continuous circulation during the reaction period. The anode and cathode plates were both composed of ruthenium–iridium plates, with a spacing of 15 cm between them. The plates were connected to a stable power supply and the cathode was aerated with an aerator (air flow 0.6 L/min). Its purpose is not only to serve as a stirring agent but also to generate hydroxyl radicals and other substances more easily in the presence of oxygen. The volume of water used at each time point was 5 L. The main body of the 3D-BER was made of a PE plate (size: L × B × H = 30 cm × 15 cm × 20 cm). The cathode was made of a pure titanium mesh, and the anode was made of Ti-IrO_2_. The filler balls had diameters of 60 mm and 90 mm and were filled with an improved polyurethane filler at a filling rate of 60%. Six aeration heads were evenly arranged at the bottom and aerators were used to provide both aeration and agitation, The effective treatment volume of the 3D-BER system was 5 L.

### 2.3. Experiment Initiation and Operation

At the beginning of the experiment, the electromagnetic Fenton system was used to pretreat the raw chemical wastewater. The goal was to utilize the strong oxidative capacity of the electromagnetic Fenton process to pretreat nitrogen-containing organic compounds such as nitrobenzene, reduce COD_Cr_, and increase BOD_5_, thereby reducing the burden on subsequent biodegradation processes. In a Fenton system, H_2_O_2_ and FeSO_4_ are often added to generate hydroxyl radicals via the release of divalent iron. In this study, iron carbon fillers were used as raw materials. Iron carbon fillers not only adsorb salt but also gradually release iron ions in different valence states. Under pH 3–3.5 conditions, Fe^0^ and Fe^2+^ were mainly released along with a reduction in the addition of conventional ferrous sulfate and a significant reduction in the generation of iron sludge. Three sets of experiments, namely, ordinary Fenton, E-Fenton, and EM-Fenton, were used as references to investigate the operating effects of the three types of Fenton systems under different experimental conditions. The HRT for each Fenton experiment was set between 2 and 12 h.

To further verify the mineralization capacity and subsequent deep treatment effects of the different Fenton systems, pretreated high-nitrogen organic chemical wastewater and domestic sewage were mixed at a ratio of 3:1 as the influent for the 3D-BER. The treatment performance was verified using a sequencing batch experiment, with the 3D-BER voltage maintained at 6.5 V. Initially, wastewater from the secondary sedimentation tank of a sewage treatment plant was used for inoculation, with an initial suspended solids concentration (MLVSS) of 4630 mg/L. During the domestication period, a low-concentration mixed wastewater with a COD_Cr_ of 400 ± 20 mg/L was used for treatment. After 24 days of domestication, the COD_Cr_ removal rate stabilized at 60 ± 5%, to prove its domestication results. A formal experiment is conducted.

### 2.4. Analytical Methods

Chemical oxygen demand (COD_Cr_) was measured using a rapid analyzer (6B-200, Changzhou, China), whereas total nitrogen (TN) was determined using ultraviolet spectrophotometry following alkaline potassium persulfate digestion (UV4802, Unico, Shanghai, China). Nitrate (NO_3_^−^-N), ammonium (NH_4_^+^-N), and nitrite (NO_2_^−^-N) concentrations were analyzed using thymol blue spectrophotometry, the nascent reagent photometric method, and the N-(1-naphthyl)-ethylenediamine colorimetric method, respectively, with a measurements performed using a Prism Light 721G spectrophotometer (Shanghai, China).

After 45 days of operation in the 3D-BER, samples were collected for further analysis. The surface morphologies of the biofilm carriers and their attached biofilms in the CG and SG were characterized using scanning electron microscopy (SEM; ZEISS Gemini 500, Oberkochen, Germany). The microbial community composition in both groups was analyzed using high-throughput sequencing. PCR amplification was conducted using primers 338F and 806R, and sequencing was performed on an Illumina MiSeq PE300 platform (Jimei Biotech, Shanghai, China).

## 3. Results and Discussion

### 3.1. Surface Characteristics of Electrodes

**Figure 2:**
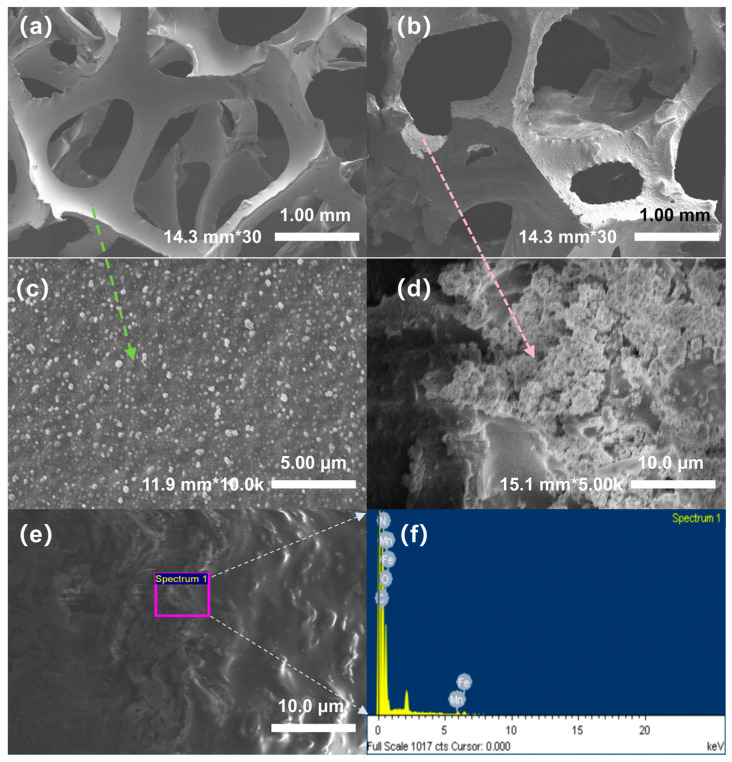
SEM surface structure and characteristics of polyurethane (**a**) unmodified, 30×; (**b**) 30 times after modification; (**c**) unmodified, 10.0K×; (**d**) modified, 5.0K×; (**e**) and (**f**) modified polyurethane energy spectrum.

To investigate the surface characteristics and elemental distribution of the modified polyurethane before and after modification, we conducted scanning electron microscopy (SEM) and energy spectrum analysis were conducted. The results revealed significant changes following modification. Figure 2 shows the SEM morphology and energy spectrum analysis results for PU fillers before and after modification. Figure 2a,c show the surface structure of the unmodified PU filler at 30× and 10.0K× magnification, respectively. The structure is observed to be a typical three-dimensional porous network with smooth pore walls and minimal surface attachments, serving as an effective physical support framework.

In contrast, Figure 2b,d show PU fillers modified with iron and manganese oxides (Fe_3_O_4_ and Mn_3_O_4_). At 30× magnification (Figure 2b), it modified PU retains a relatively intact porous structure, but the pore edges appear rough and irregular. At 5.0K× magnification (Figure 2d), a uniform distribution of numerous tiny particles across the surface of the filler is clearly visible, indicating the successful loading of iron manganese oxides on the PU skeleton. This dense particle layer not only significantly increases the specific surface area of the material but also provides more active sites for microbial attachment and electron migration [13]. Figure 2e shows an enlarged partial view of the modified PU surface, further confirming significant deposition of the substances on the surface. The EDS energy spectrum analysis of the calibration area in Figure 2e,f shows that the area contained a large number of characteristic peaks of iron (Fe) and manganese (Mn), verifying the successful loading of Fe_3_O_4_ and Mn_3_O_4_ on the surface of the PU substrate.

X-ray photoelectron spectroscopy (XPS) analysis further verified the successful immobilization of Fe and Mn oxides on the surface of PU. As shown in Figure S2a,b, the original PU carrier (corresponding to CG in Figure S2a) exhibited a simple composition containing only C, O, and N elements. In contrast, the spectrum of the modified PU filler (SG, Figure S2b) displayed distinct Fe and Mn peaks, indicating that the metal oxides were successfully introduced onto the material surface. The insets in Figure S2e,f correspond to the detailed spectra of CG, showing the absence of characteristic Fe and Mn signals, further confirming that metal loading occurred only after modification. The high-resolution Mn 2p and Fe 2p spectra of the modified sample (Figure S2c,d) revealed the valence states of the surface metals. For Mn (Figure S2c), two major peaks located at approximately 641.0 eV and 652.8 eV were assigned to Mn 2p_3/2_ and Mn 2p_1/2_. Deconvolution of the Mn 2p_3/2_ peak showed three sub-peaks at 640.3 eV, 641.3 eV, and 642.5 eV, corresponding to Mn^2+^, Mn^3+^, and Mn^4+^ species, respectively, while the Mn 2p_1/2_ region exhibited two components at 652 eV and 653 eV attributed to Mn^2+^ and Mn^3+^. This indicates the coexistence of multiple manganese oxidation states, which may enhance redox cycling and electron transfer during the bioelectrochemical process. Similarly, the Fe 2p spectrum (Figure S2d) displayed two principal peaks centered at 710.6 eV (Fe 2p_3/2_) and 724.3 eV (Fe 2p_1/2_). The fitted sub-peaks at 710.4 eV and 714.0 eV were assigned to Fe^2+^ and Fe^3+^ species, respectively, while the high-binding-energy shoulder at 724.6 eV also confirmed the presence of Fe^3+^. The coexistence of Fe^2+^/Fe^3+^ and Mn^2+^/Mn^3+^ couples suggests that the modified filler possesses abundant redox-active sites, facilitating electron mediation and catalytic regeneration of active species during operation. Compared with the XPS spectra of the unmodified carrier (Figure S2e,f), the modified PU filler clearly exhibited Fe and Mn signals, confirming the successful surface loading of Fe_3_O_4_ and Mn_3_O_4_. These findings substantiate that the modification process effectively introduced redox-active components, which are expected to enhance the electrochemical and biological denitrification performance of the 3D-BER system. The final modified filler not only retained the inherent three-dimensional porous structure of PU, but also successfully loaded a substantial amount of catalytic iron manganese oxides with good conductivity and biofilm affinity. This provides a stable and efficient reaction interface for subsequent microbial electrochemical reactions.

### 3.2. Performance of Different Fenton Pretreatment Effects

**Figure 3:**
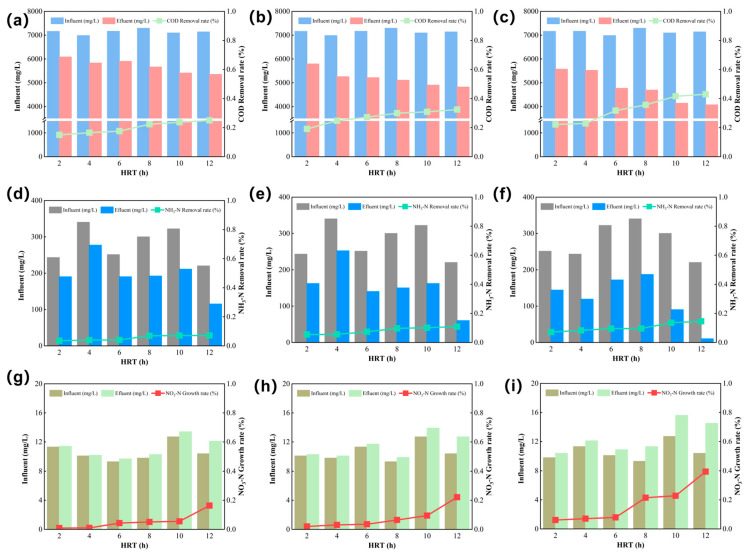
Pollutant removal efficiencies of different Fenton-based systems under various HRTs. (**a**–**c**) COD_Cr_ removal performance of the traditional Fenton (**a**), E-Fenton (**b**), and EM-Fenton (**c**) systems; (**d**–**f**) NH_4_^+^-N removal performance of the traditional Fenton (**d**), E-Fenton (**e**), and EM-Fenton (**f**) systems; (**g**–**i**) variations of NO_3_^−^-N concentration in the traditional Fenton (**g**), E-Fenton (**h**), and EM-Fenton (**i**) systems.

The pollutant removal efficiencies of the three Fenton-based systems traditional Fenton, E-Fenton, and EM-Fenton were evaluated under different HRT ranging from 2 to 12 h. Performance indicators included COD_Cr_, NH_4_^+^-N, and NO_3_^−^-N concentrations, as illustrated in Figure 3. For COD_Cr_ removal, all systems exhibited increasing efficiency with longer HRT, with the EM-Fenton process consistently achieving the highest performance. At an HRT of 12 h, COD_Cr_ removal efficiencies reached approximately 23.4% for the traditional Fenton system, 34.8% for the E-Fenton system, and 41.5% for the EM-Fenton system. This improvement was attributed to the synergistic effects of electric and magnetic fields, which enhance ·OH generation and promote continuous Fe^2+^/Fe^3+^ redox cycling [14,15].

Regarding NH_4_^+^-N removal, the traditional Fenton system showed limited effectiveness, with efficiencies generally below 4.23%. The E-Fenton system exhibited moderate improvement, particularly at longer HRTs, achieving up to 9.53% removal. The EM-Fenton system again demonstrated the best performance, with NH_4_^+^-N removal reaching nearly 14.57% at 12 h. This enhancement was associated with the more efficient utilization of ·OH radicals under electromagnetic stimulation, which promoted the oxidative degradation of nitrogen-containing pollutants.

An increasing trend in NO_3_^−^-N concentration was observed in all systems as HRT increased. This increase was mainly attributed to the oxidation of reduced nitrogen species—such as NH_4_^+^-N and organic nitrogen—into nitrate, as well as the breakdown of nitroaromatic compounds (e.g., nitrobenzene). Under strong oxidative conditions, ·OH radicals can attack both the aromatic ring and the –NO_2_ substituents of nitrobenzene, leading to ring cleavage and subsequent release of NO_3_^−^-N. This phenomenon reflects the typical transformation pathway of organic nitrogen to inorganic nitrogen during advanced oxidation processes [16].

The EM-Fenton system, owing to its enhanced radical generation, achieved deeper mineralization of nitro-organic compounds and more complete nitrogen conversion [17,18]. However, the accumulation of NO_3_^−^-N in the effluent indicates that an additional biological denitrification stage may be necessary for complete nitrogen removal. Thus, the presence of nitrate in the effluent not only suggests partial oxidation of ammonia but also indicates the effective mineralization of recalcitrant nitrogenous organics. This dual conversion pathway underscores the capability of the EM-Fenton process to decompose both inorganic and organic nitrogen species, although subsequent denitrification remains essential for total nitrogen control [19].

Previous studies have shown that magnetic fields predominantly influence the heterogeneous reactions occurring on iron surfaces, rather than altering the intrinsic homogeneous Fenton cycle. For example, Zhou et al. [20] demonstrated that a weak magnetic field can accelerate the dissolution of surface iron oxides, increase the release rate of Fe^2+^/Fe^3+^, and shorten the induction period of ZVI-based Fenton-like reactions by promoting surface corrosion. Similarly, Huang et al. [21] reported that magnetic fields can induce localized pitting corrosion at the FexOy@Fe^0^ interface, creating additional reactive sites that facilitate Fe^2+^ regeneration. These magnetic-field-driven effects provide a reasonable explanation for the enhanced Fe redox cycling and increased ·OH production observed in our EM-Fenton system. This remains a preliminary inference and will be clarified in future studies.

Overall, the EM-Fenton system demonstrated the most efficient and comprehensive pretreatment performance among the three processes, highlighting its potential to reduce organic load and enhance biodegradability in high-nitrogen chemical wastewater. A summary comparison of the three pretreatment processes is presented in Table 2 to provide a clearer overview of their COD_Cr_ removal, NH_4_^+^-N removal and NO_3_^−^-N growth efficiencies.

### 3.3. Performance of 3D-BER in Removing Pollutants, N, and P

**Figure 4:**
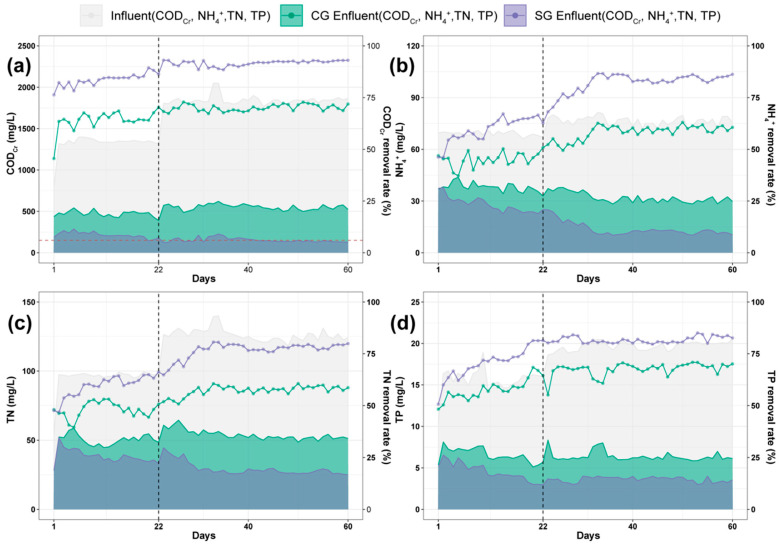
Efficiency of pollutant removal in CG and SG groups: (**a**) COD_Cr_, (**b**) NH_4_^+^-N, (**c**) TN, and (**d**) TP.

Based on previous voltage regulation experiments, the optimal operating voltage for the SG was determined to be 6.0–6.5 V, and the HRT was set to 24 h to ensure processing efficiency while also considering engineering application costs. The COD_Cr_ variation in both systems is shown in Figure 4a. In the first stage of operation, with an initial COD_Cr_ concentration of 1275 mg/L, significant differences in the removal of organic matter were observed between the CG and SG systems. During days 1–22, the average COD_Cr_ concentration in the effluent of the CG was 450.74 mg/L, with an average removal rate of 65.71%. In contrast, the COD_Cr_ concentration in the effluent of the SG was only 179.52 mg/L, with a removal rate of up to 86.34%, an increase of approximately 20.63% compared to the CG. In the second stage (days 22–60), the influent COD_Cr_ concentration was increased to 1800 mg/L. Under these conditions, the removal rate of the CG remained at 70.1%, whereas the SG maintained a high efficiency, with an effluent COD_Cr_ concentration of 142.75 mg/L, corresponding to a removal rate of 92.3%, an increase of approximately 22.2% compared to the CG. This difference was primarily owing to the role of the three-dimensional electrode system in the SG. The electrode provides a continuous and stable source of electrons, enhancing the electrochemical oxidation process, whereas the biofilm on the electrode surface forms an electroactive microenvironment, which promotes increased electron transfer rates and enzyme activity, thereby enhancing the degradation of difficult to degrade organic pollutants such as nitrobenzene [12,22].

The NH_4_^+^-N removal performance is presented in Figure 4b. In terms of NH_4_^+^-N removal, the influent concentration in the first-stage was approximately 75 mg/L, with an average removal rate of 42.7% for the CG and 59.7% for the SG, resulting in an increase of approximately 17% in removal efficiency. In the second stage, after stable system operation was achieved, the removal rate in the CG increased to approximately 60%, whereas that of the SG further increased to 84.82%. The effluent NH_4_^+^-N concentration in the SG remained at approximately 13 mg/L, an improvement of 24.82% compared with that of the CG. The superior performance of the SG was closely associated with the iron-manganese oxides loaded in its filler. Under a weak electric field stimulation, the sustained release of Fe^2+^ and Mn^2+^ only promoted microbial activity and enhanced the electron transfer within in the electrode microbe synergistic system. Moreover, Fe^2+^ can act as an electron donor in the autotrophic denitrification process, facilitating the reduction in nitrate and nitrite to nitrogen gas, thereby enhancing the overall nitrogen removal efficiency [23,24].

The TN removal performance of the two groups is shown in Figure 4c. In terms of total nitrogen (TN) removal, the initial influent TN concentration for both groups was 112.4 mg/L. There was no significant difference in removal efficiency between the CG and SG during the first 10 days of the experiment. However, from the 11th day onward, the SG exhibited a higher processing efficiency and a faster system response. The TN removal rate in the CG increased from 46.3% to 60.6% before the day 33 and then stabilized between 57.5% and 61.0%. In contrast, the SG reached a removal rate of 69.29% on the day 7 and stabilized between 77.0% and 80.2% on by day 11, with an overall removal efficiency approximately 18–22% higher than that of the CG, and achieving the stable operation approximately 4 days earlier. The fundamental reason for this difference was the effective activation of the hydrogen autotrophic denitrification mechanism in the SG. In the cathode region, Fe^0^/Fe^2+^ under low voltage can trigger the sustained release of trace amounts of H_2_, serving as an electron source for autotrophic denitrifying bacteria such as *Thiobacillus* and *Hydrogenophaga*, achieving a denitrification process driven by inorganic electron donors. At the same time, weak electrical stimulation enhances the extracellular electron transfer (EET) capability of microorganisms, further improving the efficiency of the reduction reaction during denitrification and shortening the system start-up time [25,26]. The TP removal efficiency is illustrated in Figure 4d. In terms of total phosphorus (TP) removal, the TP removal rate of the CG increased from 48.3% to 68.4% during the first stage and remained stable at 67.7% in the second stage. In contrast, the TP removal rate of the SG increased from 51.6% to 81.4%, and then stabilized at 81.1%. Compared with the CG, the SG improved the TP removal rate by approximately 13.5% and reached the stable phase approximately 10 days earlier.

The significant improvement in TP removal efficiency was attributed to the electrochemical synergistic effect of iron manganese oxides in the electrode system. Under weak electric field conditions, Fe^3+^ and PO_4_^3–^ can form insoluble FePO_4_ precipitates, thereby removing phosphorus through chemical co-precipitation. The sustained release of Mn^2+^ promotes metabolic processes in polyphosphate accumulating bacteria and enhances the efficiency of biological phosphorus removal. Finally, the SG achieved a synergistic effect of electrochemical precipitation and biological phosphorus removal, resulting in a significantly better TP removal capacity than that of traditional processes. Concurrently, our testing revealed that after the system reached stability, the dissolution of iron and manganese stabilized at 0.5 mg/L and 0.1 mg/L, respectively.

In summary, the SG exhibited superior pollutant removal capabilities across the following four indicators, COD_Cr_, NH_4_^+^-N, TN, and TP. The dual mechanism of electron and electric field stimulation through electrodes significantly enhanced the metabolic capacity of the microbial system and the overall treatment efficiency of the reactor, demonstrating strong application potential for the treatment of high-nitrogen organic chemical wastewater.

Furthermore, an economic evaluation based on a unified calculation method showed that the operating cost of the electro-Fenton section was approximately 1.5–2.0 $/kg COD_Cr_, markedly lower than the typical ~5 $/kg COD_Cr_ reported for conventional electro-Fenton processes [16]. Under the same framework, the 3D-BER operated at around 1.1–1.25 $/kg COD_Cr_, resulting in a combined system cost of 2.6–3.25 $/kg COD_Cr_. For organic chemical wastewater characterized by high organic loads, this cost range is relatively low and generally acceptable for engineering application, further supporting the feasibility of large-scale implementation.

### 3.4. Microbial Characteristic Analysis

**Figure 5:**
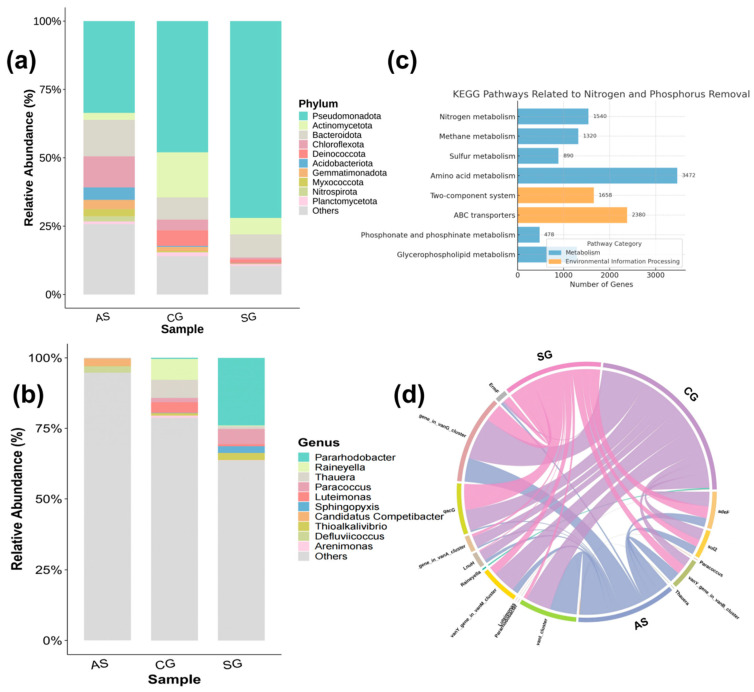
(**a**) Microbial community structure at the phylum level; (**b**) Microbial community structure at the genus level; (**c**) Functional pathway enrichment map (KEGG); (**d**) correspondence between drug-resistant genes and dominant microbial genera across different samples.

In the deep denitrification treatment of high-nitrogen organic wastewater, 3D-BER technology effectively promotes electron migration, heterotrophic autotrophic denitrification coupling, and selective enrichment of dominant functional bacterial communities by providing an electrochemical energy field and a porous carbon-iron packing interface [27]. To investigate the underlying microbial mechanisms, the microbial community evolution in the CG (non-electrified system) and SG (electrified 3D-BER system) was analyzed in comparison with the original activated sludge (AS) used for inoculation. An integrated analysis was conducted from three perspectives: microbial composition (Figure 5a,b), functional pathways (Figure 5c), and resistance gene networks (Figure 5d).

Figure 5a shows the microbial community composition characteristics of the AS, CG, and SG samples at the phylum level. The results indicate that in the SG, Pseudomonadota had a significant advantage, accounting for approximately 75%, which was significantly higher than in the CG (60%) and AS (approximately 50%). This phylum contains numerous electroactive and denitrifying genera, such as *Pseudomonas* and *Thauera*, which can achieve effective electron transfer at electrode-mediated interfaces and form the core microbial basis for electron-flow-driven denitrification reactions in the 3D-BER. Actinomycetota and Bacteroidetes also accounted for a high proportion in the SG. The former exhibits strong biological flocculation and extracellular polysaccharide synthesis functions, which contribute to the development of stable biofilm structures. The latter is involved in the degradation of complex organic matter and the release of carbon sources, providing substrate support for heterotrophic denitrification. In contrast, the AS and CG exhibited higher community diversity but less prominent dominant phyla, indicating weaker system selectivity for key functional bacteria. These findings indicate that under electrode simulation, the 3D-BER achieves targeted enrichment of specific functional categories by regulating the microenvironment, such as potential, conductivity, and redox state, and enhancing the denitrification potential and stability of the system.

Figure 5b further illustrates the microbial compositions of the three samples at the genus level. Consistent with the results at the gate level, the SG showed a stronger trend of enrichment in functional bacterial genera. The relative abundance of *Pararhodobacter* in the SG was approximately 30%, which was significantly higher than that in the CG and AS (<5%). This genus has been reported to demonstrate efficient organic matter degradation and denitrification capabilities and exhibits strong electron acceptance ability under electrode activation conditions, providing key support for heterotrophic denitrification processes in 3D-BER [28]. Similarly, *Raineyella* and *Thauera* dominated the SG with relative abundances of 20% and 12%, respectively. Both are widely recognized as key participants in enhanced denitrification systems. *Thauera* is a typical denitrifying bacterium that can use NO_3_^−^ as an electron acceptor to reduce metabolism in anaerobic or even electrically neutral environments, whereas Raineyella can metabolize complex nitrogen sources and synergistically contribute to nitrogen removal pathways [29]. Overall, the 3D-BER system induced dominant microbial communities centered around *Pararhodobacter* and *Thauera* through the electrode interface, significantly enhancing the microbial foundation of the reactor for nitrogen conversion and shock load resistance, exhibiting stronger denitrification selectivity and ecological stability.

Figure 5c presents the functional metabolic prediction results of the microbial communities in the different operating groups (at the KEGG primary pathway level). Compared with the CG and AS, the SG showed a significant increase in the relative abundance of core functional modules such as Nitrogen Metabolism, Energy Metabolism, and Membrane Transport, indicating that the three-dimensional electrode environment significantly promoted the enrichment of functional pathways related to denitrification. The relative abundance of the “nitrogen metabolism” module in the SG was approximately 2.1%, nearly twice that of the CG (0.9%) and AS (1.2%). This improvement corresponds to the efficient removal of ammonia nitrogen and total nitrogen observed in the reactor. This functional pathway includes key enzymes, such as nitrate reductase (*narGHI*), nitrite reductase (*nirS*/*nirK*), and nitric oxide reductase (*norB*), further supporting the activation of denitrification pathways dominated by Pararhodobacter and Thauera in the system. Additionally, the upregulation of energy metabolism pathways (e.g., oxidative phosphorylation) suggests that the electrode energy supply enhances microbial electron transfer efficiency, providing stronger energy support for multistep nitrogen reduction processes [30]. The enhancement of membrane transport functions facilitates the efficient exchange of substrates, metabolites, and electron shuttles, which serves as an important foundation for achieving synergistic metabolism and electron-mediated migration. The systematic improvement of these functional modules indicates that the 3D-BER system enables efficient transformation of complex pollutants in high-nitrogen wastewater through microbial community restructuring and functional enhancement.

To further investigate the differences in microbial functional group composition and drug resistance gene occurrence among the different sludge systems, a ternary string diagram was constructed to illustrate the relationships between microbial genera, samples, and resistance genes (Figure 5d). The AS was a traditional activated sludge system, the CG was a non-electrified ordinary sludge system, and the SG was an experimental group incorporating a three-dimensional biofilm electrode system. The results showed that the SG dominated the connections between microorganisms and samples. Among the dominant bacterial genera, *Pararhodobacter* (≈30%), *Raineyella* (≈20%), and *Thauera* (≈12%) showed strong associations with the SG samples, contributing most to the chord widths linked to denitrification pathways. These results are consistent with the community composition shown in Figure 5b, confirming that these genera play essential roles in maintaining the electroactive denitrifying community structure under an applied electric field.

The SG group also exhibited a strong functional association between the samples and drug resistance genes. Connections with the SG as the source node accounted for 30.5% of the total, with strong associations observed for multiple resistance genes, especially *qacG* (33.1%), *adeF* (27.6%), and the *vanY1* (25.4%). These results indicate that the microbial community in the SG has strong denitrification metabolic activity as well as high pressure resistance adaptability. In contrast, the proportion of antibiotic resistance gene connections in the AS was only 22.4%, with weaker connection strengths, such as the *vanY1* (6.8%) and *LnuH* (7.7%). This suggests that traditional sludge systems have limited functional redundancy and stability in their microbial systems when facing high toxic organic loads and resistance pressures. The CG accounted for 27.1% of the connections; although some indicators were better than in the AS group, overall, it was still lower than the SG.

In summary, the three-dimensional biofilm electrode reactor system improved the enrichment and metabolic expression of dominant denitrifying functional bacteria through electrochemical simulations, significantly improving the stress resistance and functional diversity of the microorganisms in the system [24,31]. These results support the systematic advantages of the SG group in deep nitrogen and phosphorus removal processes from a microecological structure perspective, providing theoretical support for the subsequent development of electrically assisted bioreactors with strong stability, high nitrogen removal efficiency, and superior disturbance resistance.

### 3.5. Dual Electric Joint System Processing Mechanism

**Figure 6:**
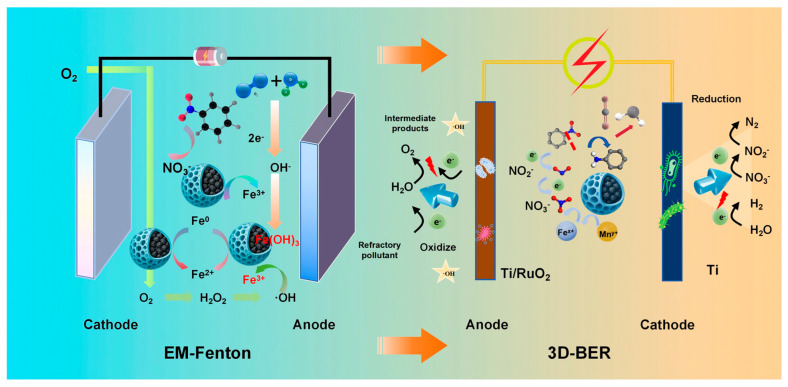
Mechanism diagram of the EM Fenton + 3D-BER dual-electric system for the combined treatment of high-nitrogen organic chemical wastewater.

The dual-electrical system, composed of EM-Fenton pretreatment and 3D-BER, exhibited a synergistic mechanism for the degradation of high-nitrogen organic chemical wastewater, as illustrated in Figure 6.

In the EM-Fenton process, iron–carbon composite fillers serve as both catalytic materials and electron donors. Under the stimulation of electric and magnetic fields, Fe^0^ is gradually oxidized to Fe^2+^ and Fe^3+^, which continuously participate in Fenton-like redox reactions, promoting sustained ·OH generation. These hydroxyl radicals efficiently attack refractory nitrogenous organic pollutants such as nitrobenzene, resulting in ring opening and the oxidative transformation of -NO_2_ into NO_3_^−^. This process reduces COD_Cr_ and NH_4_^+^-N concentrations and also facilitates the conversion of organic nitrogen into inorganic nitrate, representing a key feature of advanced oxidation pathways. Moreover, because of the controlled release of iron species from the filler, the EM-Fenton system significantly reduces the demand for external Fe^2+^ dosing and minimizes secondary iron sludge production.

Following pretreatment, the effluent enters the 3D-BER system, where further removal of carbon, nitrogen, and phosphorus occurs. The modified PU filler, loaded with Fe_3_O_4_ and Mn_3_O_4_, contributes to enhanced microbial colonization and electroactivity owing to its increased surface roughness and abundant catalytic sites. Within the cathodic zone of the 3D-BER, residual Fe^2+^ and Mn^2+^ from the EM-Fenton stage continue to act as electron donors, facilitating the formation of electron shuttles and promoting EET in denitrifying biofilms [32,33]. In particular, trace H_2_ generation under low-voltage conditions provides a stable inorganic electron source, effectively activating the autotrophic denitrification pathways mediated by hydrogenotrophic bacteria (e.g., Thiobacillus and Hydrogenophaga).

The synergistic application of the EM-Fenton and 3D-BER system shortens the start-up time and enhances the nitrogen conversion efficiency by coupling advanced oxidation with bioelectrochemical denitrification. Although this integrated strategy requires additional energy input, the simplification of unit processes and an increase in treatment efficacy offer overall cost-effective advantages. Notably, the dual-electrical approach improves the biodegradability of the influent through front-end oxidation and enables precise electron distribution in the back-end biological system, thereby facilitating the high-rate removal of COD, NH_4_^+^-N, TN, and TP in a single streamlined process.

In summary, the EM-Fenton + 3D-BER dual-electrical system is a highly effective strategy for treating high-nitrogen-refractory wastewater. This system integrates chemical oxidation with bioelectrochemical reduction, minimizes reagent consumption and sludge generation, and maximizes pollutant degradation through targeted electron management. This coupling mechanism provides both theoretical and practical guidance for the engineering and design of future electrically assisted advanced treatment systems for chemical wastewater.

## 4. Conclusions

In this study, a dual-electrical treatment system combining the EM-Fenton pretreatment with 3D-BER was developed to efficiently remove pollutants from high-nitrogen organic chemical wastewater. The EM-Fenton stage effectively reduced COD_Cr_ and NH_4_^+^-N levels and facilitated the conversion of organic nitrogen from nitrobenzene into NO_3_^−^-N through ·OH-induced oxidative degradation. Compared with conventional Fenton processes, it minimized Fe^2+^ dosing and reduced iron sludge generation.

Following pretreatment, the 3D-BER system, equipped with Fe_3_O_4_ and Mn_3_O_4_ modified polyurethane fillers, achieved enhanced removal of COD_Cr_, NH_4_^+^-N, TN, and TP. This improvement was attributed to improved electron transfer, the formation of an electroactive microbial environment, and the activation of autotrophic denitrification via trace H_2_ release at the cathode. The dual-electrical configuration also shortened the system start-up time and improved microbial selectivity for key denitrifying genera such as *Pararhodobacter* and *Thauera*.

Overall, the coupled EM-Fenton and 3D-BER systems integrate chemical oxidation with bioelectrochemical reduction, offering a cost-effective and highly efficient strategy for the treatment of complex, nitrogen-rich wastewater. This study provides valuable technical guidance for the development of advanced, electrically assisted wastewater treatment processes.

## Figures and Tables

**Table 1:** Water quality characteristics of nitrobenzene wastewater.

Water Quality Indicators	COD_Cr_ (mg/L)	pH	TDS (g/L)	TN (mg/L)	NH_4_^+^-N (mg/L)	TP (mg/L)	Fe (mg/L)
Raw water (EM-Fenton influent)	7000 ± 200	10 ± 1	5 ± 0.5	300 ± 50	250 ± 50	14 ± 5	14 ± 2
3D-BER influent	1600 ± 200	8 ± 1	5 ± 0.5	121 ± 5	71 ± 5	14 ± 5	14 ± 2

**Table 2:** Comparison of pollutant removal efficiencies among the EM-Fenton, E-Fenton and Fenton processes.

Methods	COD_Cr_ Removal Efficiency (%)	NH_4_^+^-N Removal Efficiency (%)	NO_3_^−^-N Growth Efficiency (%)	HRT (h)
EM-Fenton	41.5	14.57	39.42	12
E-Fenton	34.8	9.53	22.11	12
Fenton	23.4	4.23	18.35	12

## Data Availability

The original contributions presented in this study are included in the article/Supplementary Materials. Further inquiries can be directed to the corresponding author.

## Supplementary Materials

The following supporting information can be downloaded at: https://www.mdpi.com/article/10.3390/toxics13121059/s1, Figure S1. Schematic diagram of the arrangement of the rubidium permanent magnets around the EM-Fenton reactor. Figure S2: XPS spectra of the modified polyurethane filler (SG) and control group (CG).
