# Supplementary material for: Electromagnetic Fenton Combined with Electro-Biological Coupling Technology for Treating High-Nitrogen Organic Chemical Wastewater
Source: Toxics. 2025 Dec 6;13(12):1059. doi: 10.3390/toxics13121059 (PMC12737508; doi:10.3390/toxics13121059)
Supplement: Supplementary file 1 [file toxics-13-01059-s001.zip › toxics-4009744-supplementary.pdf]

Electromagnetic Fenton combined with electro biological coupling technology for treating high nitrogen organic chemical wastewater

Dengyan Mu[a,b],Xiaojie Chen[c], Peiyu Zhao[b], Houhui Zhang [d],Zhujun Bai[b],Baoshan Wang[b*]

[a]*Gansu Youyuan Environmental Protection Engineering Technology Co., Ltd 730030, PR China.*

[b]*School of Environmental and Municipal Engineering, Lanzhou Jiaotong University, Lanzhou 730070, PR China*

[c]*Shaanxi Key Laboratory of Environmental Engineering, Xian University of Architecture and Technology, Xian, 710055, PR China*

[d]*Gansu Appraisal Center for Ecological Environment&Engineering, Lanzhou 730000, PR China*

[*]**Corresponding author:** Baoshan Wang

Tel.:+86 13919126540 Fax: +86 0931 4956017

E-mail: wbs@mail.lzjtu.cn

Postal Address: School of Environmental and Municipal Engineering, Lanzhou Jiaotong University, Lanzhou 730070, PR China.

Figure S1. Schematic diagram of the arrangement of the rubidium permanent magnets around the EM-Fenton reactor.

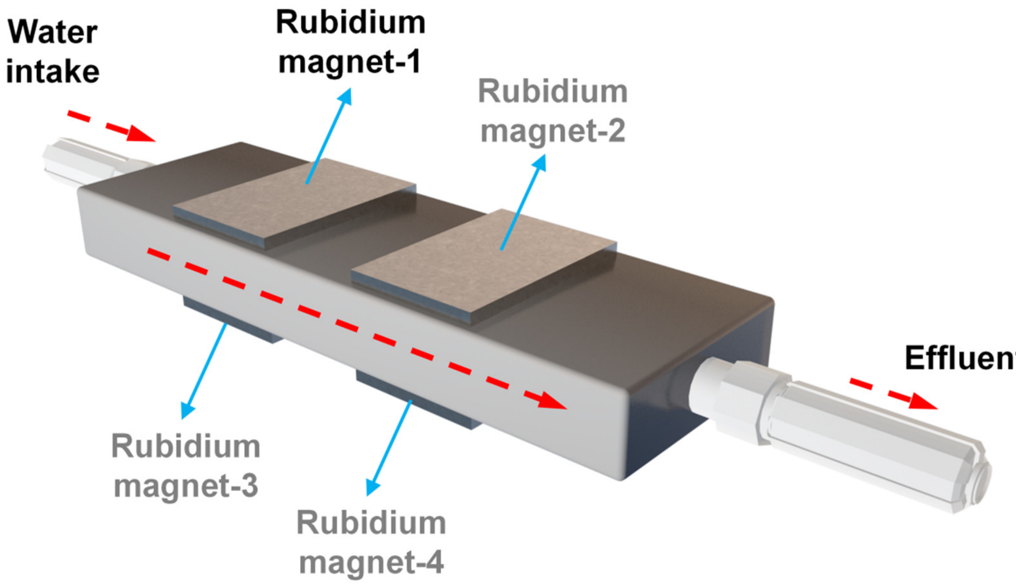

Figure S1. Schematic diagram of the arrangement of the rubidium permanent magnets around the EM-Fenton reactor. (Magnets-1 and 2 are placed on the top surface of the rectangular reactor, while magnets-3 and 4 are fixed directly opposite on the bottom surface. The two vertically aligned magnet pairs form a closed magnetic circuit across the reactor wall, so that the magnetic flux density at the inner reactor wall is maintained at approximately 50 mT (±10 mT) along the water flow path from the inlet to the effluent).

Figure S2.XPS spectra of the modified polyurethane filler (SG) and control group (CG).

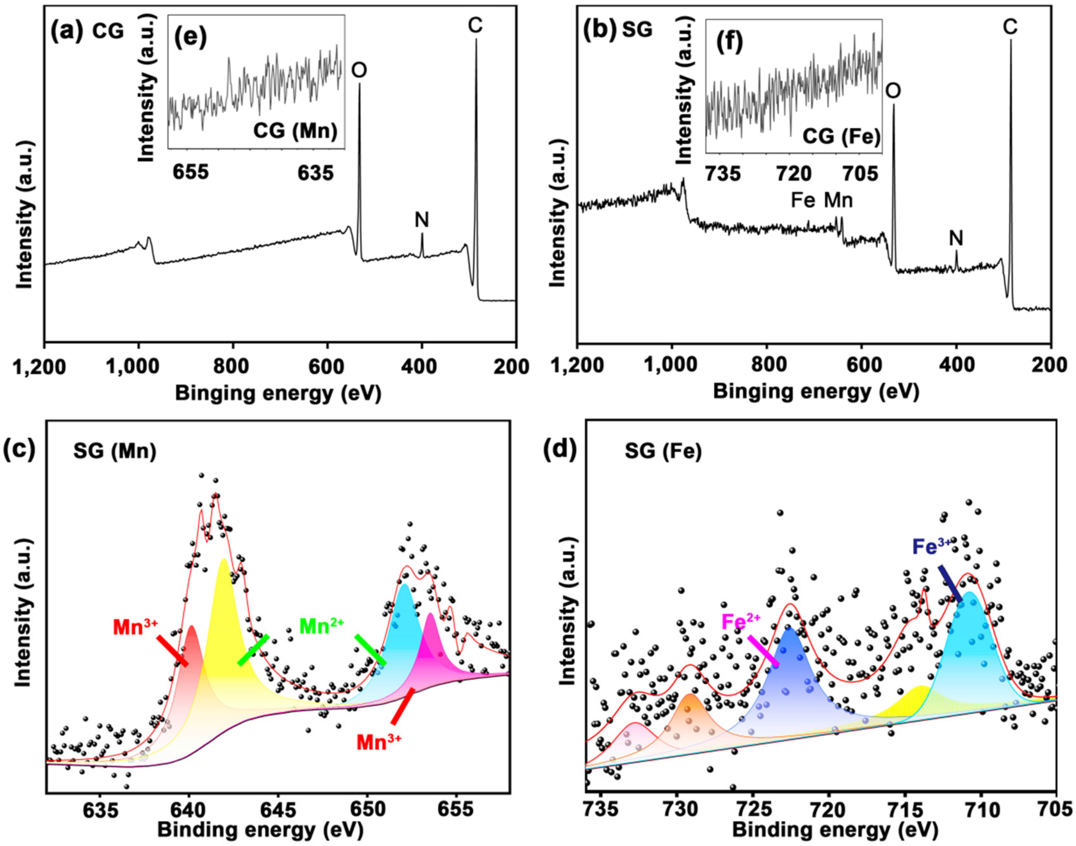

Figure S2. (a) Full-spectrum of CG; (b) Full-spectrum of SG; (c) High-resolution Mn 2p spectrum of SG; (d) High-resolution Fe 2p spectrum of SG; (e) Mn spectrum of CG; (f) Fe spectrum of CG. .(e) Mn spectrum of CG and (f) Fe spectrum of CG are shown as insets in (a) and (b)
